# Septic Obturation of a Knee Endoprosthesis Caused by *Aspergillus clavatus*

**DOI:** 10.3390/pathogens12101270

**Published:** 2023-10-23

**Authors:** Robert Kuthan, Gabriel Lawrence Zaremba-Wróblewski, Flynn Ott, Dorsa Soltaninia

**Affiliations:** 1 Chair and Department of Medical Microbiology, Medical University of Warsaw, 02-004 Warsaw, Poland; gabrielzaremba@gmail.com; 2 Student Scientific Club Microbiology Applied to Clinics and Real Life for Students (MACR-S) Affiliated to Chair and Department of Medical Microbiology, Medical University of Warsaw, 02-091 Warsaw, Poland

**Keywords:** *Aspergillus*, *Aspergillus clavatus*, infection, prosthetic joint infection (PJI)

## Abstract

*Aspergillus clavatus* is a rare opportunistic fungal pathogen that can be isolated from various environmental sources, including soil and animal feces. Although infrequent, infections caused by *A. clavatus* can be severe in immunocompromised patients. Here, we present a case of a prosthetic joint infection (PJI) in a 74-year-old female patient caused by *A. clavatus*. The patient presented with left knee pain, and septic loosening of the left knee endoprosthesis was diagnosed. She underwent surgical revision with the implantation of an antibiotic spacer and microbiologic testing. The results came back positive for both *Staphylococcus lugdunensis* and *A. clavatus* (which is found in only a fraction of a percent of PJIs). She was treated with oral antimicrobials for 3 months postoperatively. This case report vividly illustrates a clinical scenario that underscores the significance of rigorous microbiologic testing procedures, accurate pathogen identification, unwavering vigilance in testing protocols, and a cautious approach that avoids succumbing to the seductive simplicity of Occam’s razor.

## 1. Introduction

As the global population ages and medical advancements continue to be developed, patient quality-adjusted life years continue to improve. One specific field that is rapidly developing is endoprosthetic joint replacements. With this large field, aimed at elderly patients, rapidly expanding, along with other fields such as transplantology, we are seeing an increasing number of patients that are to some degree immunocompromised (whether from iatrogenic or natural causes) undergoing major operations. While fungal infections have been quickly overshadowed in everyday situations by their ever-present bacterial counterparts, immunocompromised patients are the ideal environment for fungal infections to develop. These changes require medical professionals to continuously expand their diagnostics abilities, remembering alternative causes of infections. Fungal prosthetic joint infections (PJIs) remain a poorly understood and underappreciated entity. Fungal PJIs are characterized by fungal invasion of prosthetic components and their surrounding tissues. Their symptoms can often be mild, eluding detection for quite some time, or simply being misinterpreted as a more common culprit. PJIs are frequently of a bacterial origin, usually coagulase-negative staphylococci, but we are increasingly recognizing the role played by the rare etiologies like unicellular fungi, such as *Candida* spp., and even rarer etiologies like filamentous fungi, such as those from the *Aspergillus* genus.

Aspergilli rank among the most prevalent fungi worldwide. They adopt a saprotrophic lifestyle (decomposers), which makes them significant contributors to food and feed spoilage. Furthermore, aspergilli can opportunistically infect plants, animals, and most importantly humans.

Within the *Aspergillus* genus, approximately 40–50 species have been associated with human infections, with the most common ones being *A. fumigatus, A. flavus, A. terreus,* and *A. niger*.

Aspergillus clavatus is taxonomically classified as follows: Kingdom: Fungi, Phylum: *Ascomycota*, Class: *Eurotiomycetes*, Order: *Eurotiales*, Family: *Aspergillaceae*, Genus: *Aspergillus*, Species: *Aspergillus clavatus*.

*A. clavatus* is distributed globally in the environment as a saprophyte, particularly in tropical, subtropical, and Mediterranean regions.

From a medical perspective, it is important to note that while *A. clavatus* is considered an infrequent causative agent of mycoses in humans, it has been implicated in specific cases such as endocarditis, extrinsic allergic alveolitis, and mycotoxicoses in animals due to its production of various mycotoxins, including patulin, cytochalasin E, tryptoquivalines, ascladiol, and ribotoxins. Due to its low prevalence in infections and the limited research focused on *A. clavatus* susceptibility to antifungal medications, relatively little is known about its response to antimycotic treatments.

This case describing a 74-year-old patient with a fungal septic obturation of a knee endoprosthesis perfectly illustrates the types of challenges medical professionals will be facing more and more frequently in the coming years.

## 2. Materials and Methods

### 2.1. Culture

Clinical specimens (5 samples taken intraoperatively, before implementation of an antibiotic spacer) were aseptically homogenized and cultured on classical liquid media used for fungi and bacteria (Sabouraud broth and Schaedler broth with vitamin K1) and incubated at 30 °C and 37 °C, respectively.

Once the Schaedler broth turned turbid, the material was subcultured onto Columbia agar with 5% sheep blood, Schaedler agar with 5% sheep blood and vitamin K1, and chocolate agar. Colonies were detected and subjected to identification using a Matrix-Assisted Laser Desorption/Ionization Time-of-Flight Mass Spectrometer (MALDI-TOF MS).

**Figure 1:**
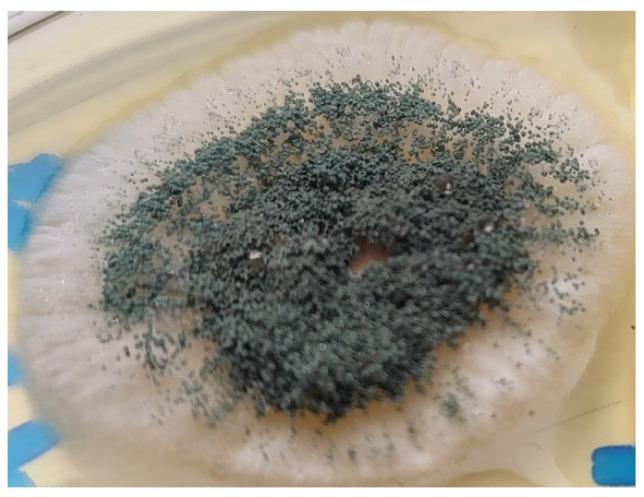
*A. clavatus* culture on Sabouraud agar.

**Figure 2:**
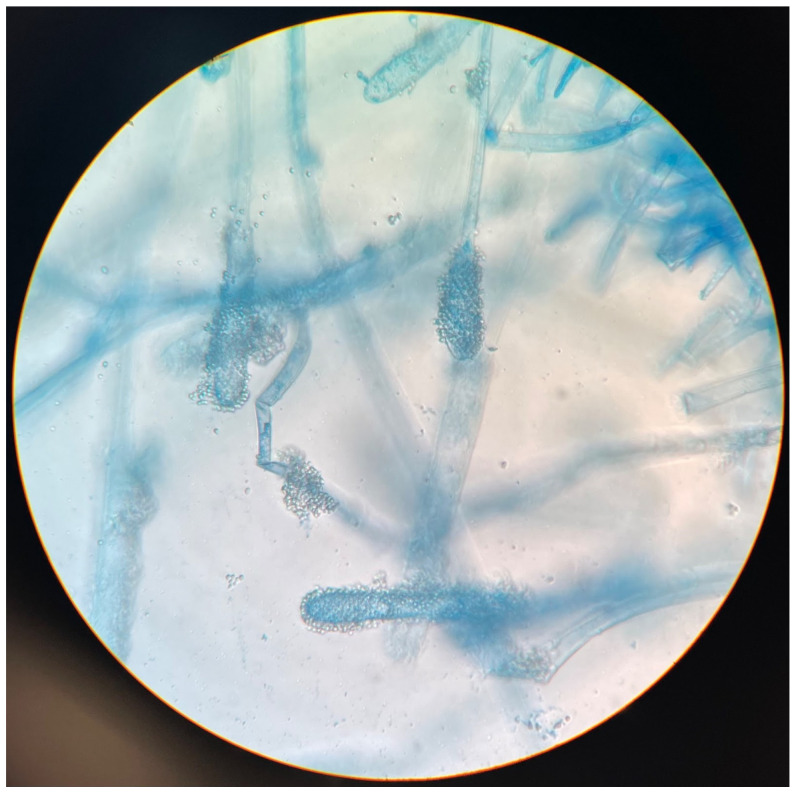
Clavate-shaped conidiophores of *A. clavatus* at 400× magnification, lactophenol cotton blue wet mount preparation.

**Figure 3:**
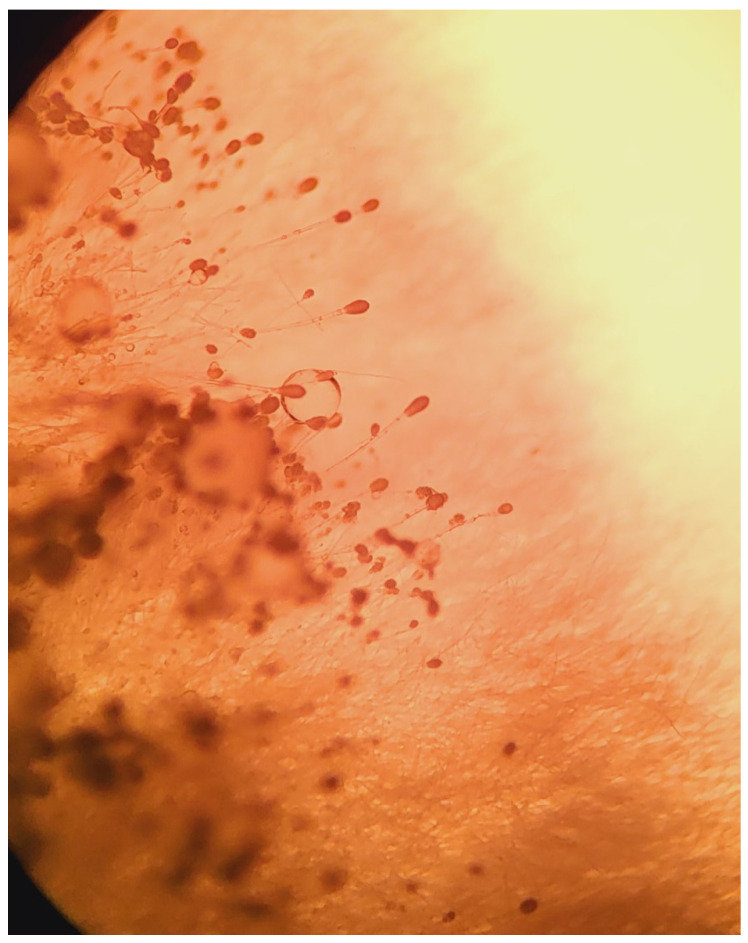
Hyphae and conidiophores of *A. clavatus* (50× magnification).

Once the Sabouraud broth turned turbid, the material was subcultured onto Sabouraud agar with gentamicin and chloramphenicol. After 3 days, growth was detected. Aerial mycelia (Figure 1) were subjected to identification based on the detailed analysis of colony pigmentation, morphology of the hyphae, and the shape/size of conidiophores after being stained with lactophenol cotton blue, and observed under a light microscope at a 400-time magnification (Figure 2) using an Olympus CH20BMF200 microscope. The microscopic analysis revealed characteristic clavate-shaped conidiophores (Figure 3).

Samples were also subjected to identification via MALDI-TOF MS. The analysis was performed with the application of the VITEK^®^ MS (bioMérieux; database version 3.0) system.

### 2.2. Drug Susceptibility

The drug susceptibility tests were performed with the MIC method (EUCAST standardized broth microdilution method). The MIC values were determined for voriconazole, itraconazole, and amphotericin B. For interpretation of the MICs, EUCAST protocols were used.

### 2.3. Results

The fungal pathogen responsible for infection was susceptible to voriconazole (0.50 mg/L), susceptible to itraconazole (0.50 mg/L), and susceptible to amphotericin B (0.25 mg/L).

## 3. Case Presentation

**Figure 4:**
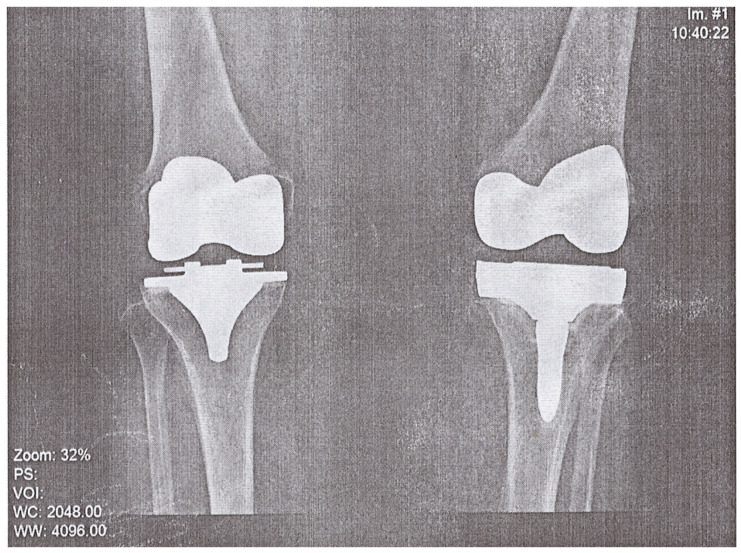
AP radiography, pre-operative, of suspected left knee PJI.

**Figure 5:**
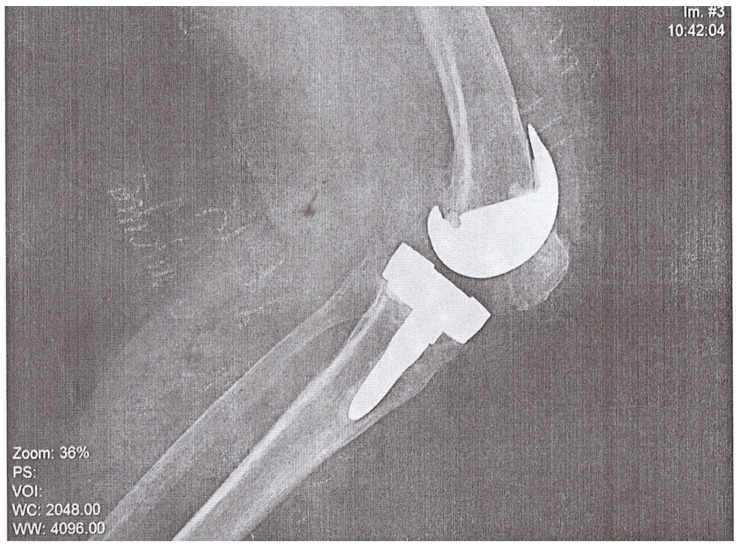
Lateral radiography, pre-operative, of suspected left knee PJI.

A 74-year-old woman presented to the Emergency Department of a university hospital with left knee pain for the past 2 months. The patient’s medical history revealed a previous left knee alloplasty (3 years prior) with a Stryker Triathlon endoprosthesis, bilateral gonarthrosis, left bundle branch block, chronic atrial fibrillation, an ischemic cerebral infarction (1 year prior), and a right knee alloplasty. Blood results showed no abnormalities other than an elevated neutrophil and monocyte percent (74.2% and 8.3%, respectively) and an elevated CRP of 106.79 mg/L. Septic obturation of the left knee endoprosthesis was diagnosed based on clinical presentation, blood work, and radiography findings (Figure 4 and Figure 5), leading to a re-alloplasty of the left knee.

This included removal of the loosened Triathlon endoprosthesis, collection of microbiologic samples, joint debridement, pulse lavage (containing an antiseptic and gentamicin), and implantation of an antibiotic spacer (gentamicin and vancomycin).

Microbiological testing was performed on samples taken from five different locations (1. Fluid from joint cavity; 2. Granulation tissue present on the low friction bearing; 3. The terminal portion of the femur; 4. Tibial bone marrow cavity; and 5. Granulation tissue present on the internal surface of the joint cavity). Microbiologic studies were positive for methicillin-sensitive *Staphylococcus lugdunensis* in samples 1 and 2 and azole-sensitive *A. clavatus* in samples 3 and 4. No samples were collected for histopathological examination since it is not required in the course of prosthetic joint infection treatment.

According to the European Bone and Joint Infection Society’s (EBJIS) definition of PJI, the isolation of an uncommon or highly virulent organism on only one sample should be considered a probable infection [1].

The patient was treated using antibiotic therapy (vancomycin at 1g i.v. intraoperatively followed by 2 g every subsequent day in the hospital with serum concentration monitoring, rifampicin at 2 × 300 mg p.o. for the 6-day stay in the hospital, thromboprophylaxis (enoxaparin), and analgesics (paracetamol, ketoprofen, metamizole).

The patient made a full recovery without any postoperative complications and was discharged with post-op recommendations including weight reduction, follow-up visits at the orthopedics outpatient clinic, and prescriptions for clindamycin at 4 × 300 mg p.o. and rifampicin at 2 × 300 mg p.o. for 3 months and itraconazole at 2 × 200 mg p.o. for 2 months.

## 4. Discussion

A significant issue regarding the diagnostics and appropriate treatment of PJIs caused by the *Aspergillus* genus is a lack of guidelines detailing fungal PJI management. The vast majority of guidelines about PJIs focus on aerobic and anaerobic bacteria. Fungal PJIs account for less than 1% of PJIs (15–20% of which are concomitant bacterial infections), with *Candida* species present in around 80% of fungal PJIs [2]. An analysis of recent publications shows that there are only 10 documented cases of *Aspergillus* spp. causing PJIs (according to a 2021 systematic literature review), 4 of which were caused by *A. fumigatus*, 3 caused by *A. terreus*, 2 caused by *A. niger*, and 1 caused by *Aspergillus* sp. [3].

Since *A. clavatus* infections occur significantly less often, there are no comprehensive antimycotic resistance maps and this is demonstrated with the lack of specific data about *A. clavatus* in the EUCAST guidelines. Therefore, from an academic standpoint, the ideal treatment for our patient would have been voriconazole. It serves as a second-generation triazole that has higher potency against *Aspergillus*, and other fungi, compared to other azoles and therefore is recommended as first-line treatment for Invasive Aspergillosis [4,5]. After considering the cost of voriconazole treatment, and the fact that this was a non-systemic infection, there was no significant benefit to using voriconazole over itraconazole [6,7].

**Figure 6:**
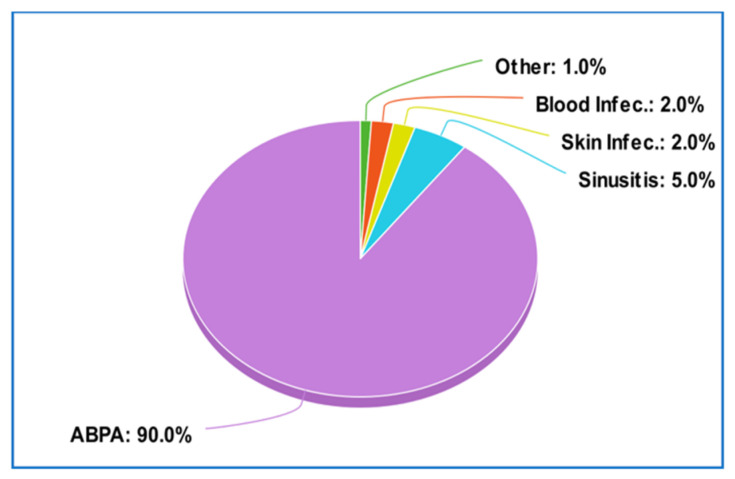
Maladies caused by *Aspergillus* spp.

The Aspergillus genus typically causes a condition known as Allergic Bronchopulmonary Aspergillosis (ABPA) in approximately 90% of cases, followed by sinusitis (5.0%), and Invasive Aspergillosis (2.0%). Infections of endoprosthetics account for a small portion of the 1% classified under the “Other” category [8] (Figure 6).

*A. clavatus* is a relatively rare pathogenic species, less often isolated from environmental samples compared to other *Aspergillus* species [7]. Risk factors associated with *A. clavatus* infection are not well defined but may include immunosuppression, prior fungal infections, and exposure to environmental sources of the fungus [7]. The majority of the literature describes *A. clavatus* as a source of mycotoxins in foodstuffs, feed-related toxicity in animals, the causative agent in “malt worker’s lung”, and individual cases of infections: dystrophic onychomycosis, endocarditis, and otomycosis [9,10,11,12,13].

**Figure 7:**
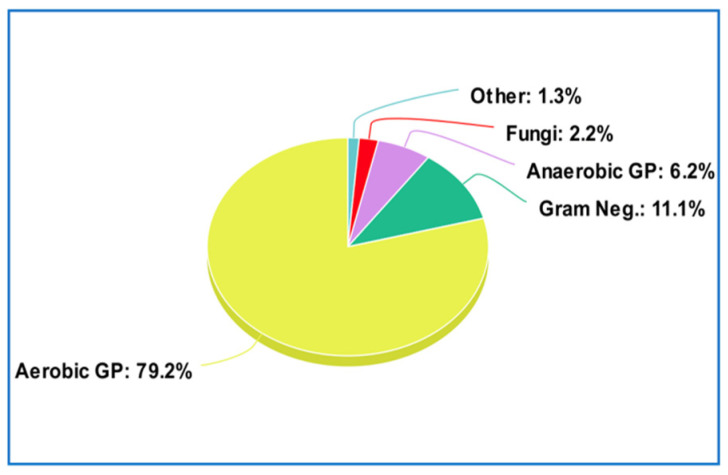
Etiologies of periprosthetic joint infections.

Prosthetic joint infections are most commonly caused by Gram-positive bacteria (79.2% of cases) with the most common including coagulase-negative staphylococci (48.56%), *Staphylococcus aureus* (12.78%), and *Enterococcus faecalis* (7.03%), followed by Gram-negative bacteria at 11.1%, anaerobic Gram-positive bacteria at 6.2%, fungi at 2.2%, and others at 1.3% [8] (Figure 7).

As a result of the pathogens that are typically found to cause PJIs, antibiotic spacers containing vancomycin and gentamicin are a mainstay in the treatment of PJIs. However, since fungal etiologies of PJIs are so rare, the standard treatment does not include the addition of antimycotic agents.

Diagnostic criteria for fungal PJI include the presence of fungal elements in histological specimens, positive fungal cultures, and radiographic findings consistent with a PJI. The diagnosis combines the aforementioned findings, along with the clinical presentation. Treatment typically involves antifungal therapy, surgical debridement, and the removal of implanted components.

According to Riaz et al. [14], certain risk factors increase the likelihood of developing a fungal PJI. Antimicrobial therapy can be received within the 3 months preceding a PJI (3.4-fold risk increase) including having a wound debridement accompanied with prolonged broad-spectrum antibiotic treatment lasting more than 5 days (7.4-fold risk increase).

## 5. Conclusions

The demand for endoprosthetics drives a large part of the medical device industry and has contributed to it a whopping USD 33.5 billion in 2022. This amount is only expected to increase in the coming years as a result of our aging population and a push towards a higher amount of quality-adjusted life years [15]. Most prosthetic infections are of a bacterial etiology, but a fungal etiology should not be dismissed. In this case, a rare fungal etiology (*A. clavatus)* was found to be the causative agent of a PJI.

Although *A. clavatus* is distributed worldwide, with particularly high concentrations in tropical and subtropical soils, and is regularly inhaled and cleared by immunocompetent patients, the fact that this endoprosthetic loosening had such copious growth of a fungus that commonly causes pulmonary infections requires attention and warrants a retrospective analysis of the diagnostic and treatment methods.

Mycological testing should be a mainstay when diagnosing patients with septic loosening of an endoprosthesis since empiric treatment does not cover fungal etiologies. An increase in vigilance and testing could lead to a reduction in the amount of missed fungal PJIs.

## Data Availability

No new data were created or analyzed in this study. Data sharing is not applicable to this article.
